# Supplementary material for: Investigating mortality salience as a potential causal influence and moderator of responses to laboratory pain
Source: PeerJ. 2024 Apr 3;12:e17204. doi: 10.7717/peerj.17204 (PMC10998629; doi:10.7717/peerj.17204)
Supplement: Supplemental Information 2 [file peerj-12-17204-s002.docx]

Ethnicity:

0: Minority

1: Han Ethnicity

Religion status:

0: No

1: Yes

Relationship Status:

0: Single

1: Non-Single

This codebook provides a detailed mapping between the numerical codes and their corresponding categorical meanings, to ensure an accurate understanding of the data's structure and content.
